# Discrete event simulation modelling for an improved patient flow at the Emergency Department, Sygehus Lillebælt, Kolding

**DOI:** 10.1186/1757-7241-20-S2-P14

**Published:** 2012-04-16

**Authors:** Dawid Kozlowski, Christian Backer Mogensen, Niels Christian Petersen

**Affiliations:** 1 Institute of Public Health, Research Unit of Health Economics, University of Southern Denmark, Denmark; 2 Emergency Department, Kolding Hospital, Denmark

## Background

The process of ongoing centralization of acute hospitals in Denmark enforces a number of challenges to be addressed. Staff levels must be adjusted not only in order to fulfil the new rules for specialists’ presence at the Emergency Department (ED), but also to meet the imposed time requirements regarding the first patient contact with a specialist along with the requirement of making an action diagnosis within 4 hours after arrival. Patient arrivals and pathways are grouped in accordance with the so called Standardized Time-based Patient Pathways (STPs) under development.

The main goal of the project is the development of an analytical tool designed to facilitate qualified decision-making with respect to the dimensioning of the ED with respect to e.g. staffing in view of the imposed time requirements.

## Methods

A Discrete Event Simulation (DES) model is developed for analysis of the flow of patients through the ED. The model offers the possibility for characterizing a number of relevant performance measures, including probabilities for the average patient in each STP for fulfilment of the imposed time requirements. The model gives rise to a number of advanced What-If analyses including adjustments in volume of staff along with changes in the clinical composition of staff without interference in the underlying real system.

## Results

The project is in the final stage of a real data implementation, and preliminary results will be reported. The model is developed in the context of Sygehus Lillebælt.

## Conclusion

The DES model is designed as a generic model framework thus amplifying the usefulness of DES in conducting comprehensive patient flow analyses at any department with emergency patients with the aim of supporting constant enhancement of the quality of patient care, patient satisfaction and effective capacity management to be brought about by an improved patient flow.

